# Executive Function and Social Cognition Performance Predicts Social Difficulty for Autistic Adults

**DOI:** 10.1002/aur.70090

**Published:** 2025-08-01

**Authors:** T. R. Wong, K. A. Boulton, E. A. Demetriou, E. E. Thomas, N. L. Phillips, L. Hankin, S. H. Park, I. B. Hickie, A. J. Guastella

**Affiliations:** ^1^ Clinic for Autism and Neurodevelopment Research, Brain and Mind Centre, Children's Hospital Westmead Clinical School Faculty of Medicine and Health, University of Sydney Sydney Australia; ^2^ Child Neurodevelopment and Mental Health Team, Brain and Mind Centre University of Sydney Sydney Australia

## Abstract

There has been limited research aimed at understanding the cognitive features that predict outcomes in autistic adults. Difficulties in social cognition and executive function (EF) processes have been proposed as important cognitive components underlying social functioning outcomes. In this study, 305 autistic adults were administered a battery of social cognition, EF, and social functioning assessments to determine the degree to which social cognition and EF factors predicted outcomes. For social cognition, hierarchical regressions showed that EF explained neither theory of mind scores nor emotion recognition scores. We then ran several mediation models to determine whether EF explained social functioning independently of social cognition. These exploratory analyses show that poorer performance‐based EF and social cognition both predicted more clinician‐observed social challenges, while poorer self‐reported EF and social cognition predicted more self‐reported social challenges. Effects on outcomes were independent of each other. Our results highlight the potential of bot EF and social cognition measures to provide clinically meaningful markers for social functioning, but via separate pathways. This study supports the utility of targeting EF and social cognition processes in autistic adults in assessment and support pathways.

**Summary**

- Some autistic adults describe having trouble understanding social situations, like understanding emotions and figuring out what others are thinking.
- They can also have difficulty with executive skills, like switching tasks or sustaining attention.
- Three hundred and five autistic adults took these tests to see how such cognitive capacities were associzated with social responsiveness.
- We found that people who had trouble with executive skills and social cognition were more likely to have problems with social responsiveness.
- These skills could have an important role in determining social functioning for autistic people.

## 1 Introduction

Autism is partly characterized by the presence of challenges in social communication and interaction (American Psychiatric Association 2013; Volkmar et al. 2004) that contribute to poorer psychological and functional outcomes (De Vries and Geurts 2015). While both cognitive and social factors are believed to contribute to these difficulties, the majority of this research has been conducted in children. The transition into adulthood is marked by new challenges, social roles, and environments. These challenges are further compounded by the absence of effective supports which has been referred to as the ‘services cliff’ (Bemmer et al. 2021). As autistic adults mature out of childhood support networks, there is an absence of both knowledge and services to effectively support better outcomes. There is, therefore, an urgent need to better understand factors that contribute to social difficulties in autistic adults and to develop supports that can address these needs.

There has been extensive investigation into the cognitive factors that underpin and contribute to social functioning difficulties. Poorer *social cognition* has long been thought to underlie observed social functioning difficulties. Social cognition refers broadly to the processes involved in perceiving, storing, processing, and interpreting information about oneself and others (Green et al. 2015). These processes are sometimes broken down into lower‐ and higher‐order cognitive processes. While lower‐order social cognitive processes are exercised intuitively and at the perceptual level, higher‐order social cognitive processes are conscious, effortful, and facilitate the interpretation of social information (see Mitchell and Phillips 2015 for a discussion). Emotion recognition (ER) is one aspect of lower‐order social cognition, while theory of mind (ToM; the capacity to infer others' thoughts and feelings) is an aspect of higher‐order social cognition (Pepper et al. 2018). Autistic individuals have been found to face challenges in exercising both higher‐ and lower‐levels of social cognition, including recognizing, matching, and labeling facial emotions (Rump et al. 2009; Peñuelas‐Calvo et al. 2019). Similarly, autistic adults can often find it difficult to interpret complex social events such as jokes, sarcasm, and faux pas (Baron‐Cohen et al. 1999; Kelly et al. 2021).

In addition to social cognition, there has been extensive investigation into the role of Executive Functioning (EF) in autistic individuals. EF refers to a spectrum of higher‐order mental processes that enable and regulate goal‐directed behaviors (Demetriou et al. 2019). EFs are traditionally assessed using standardized, performance‐based assessments taken under timed conditions. These tests reveal that autistic individuals show reduced performance across multiple executive domains, such as cognitive flexibility, inhibition, planning, and verbal fluency (Geurts et al. 2014; Russo et al. 2007; Hill 2004) that persist across the lifespan (De Vries and Geurts 2015; Bemmer et al. 2021; Green et al. 2015; Mitchell and Phillips 2015; Pepper et al. 2018; Rump et al. 2009; Peñuelas‐Calvo et al. 2019; Baron‐Cohen et al. 1999; Kelly et al. 2021; Demetriou et al. 2019; Geurts et al. 2014; Russo et al. 2007; Hill 2004). Behavioral rating‐based scales have also been used to assess EF functions in standardized environments and further indicate how EF difficulties impact daily functioning for autistic adults (Kenworthy et al. 2008; Demetriou, Lampit, et al. 2018; Leung and Zakzanis 2014).

While many have studied the role of these cognitive processes, most of this research utilizes a group differences approach by comparing autistic and neurotypical groups on test performance of these measures separately (Pellicano 2012; Joseph and Tager–Flusberg 2004). This precludes the study of variability within autistic populations and how cognitive processes such as EF and social cognition predict outcomes (Lai et al. 2014; Masi et al. 2017). Studies of autistic children continue to heavily outnumber studies conducted in adults.

Researchers have long discussed the relation between EF and social cognition, given that both abilities mature at similar times during typical development (Wade et al. 2018). Having better executive skills at baseline predicts better theory of mind (ToM) ability at a later time in neurotypical toddlers (Hughes and Ensor 2007; Müller et al. 2012), pre‐schoolers (Hughes 1998; Marcovitch et al. 2015) and primary school children (Austin et al. 2014; Lecce et al. 2017). There is preliminary evidence for this association in autistic children. Administering several ToM and performance‐based EF tests, Pellicano (Pellicano 2007) found that 4–7 year‐old autistic children could demonstrate intact EF (planning, set‐shifting and inhibition) in the presence of an impaired ToM (unexpected transfer of items). This suggests that executive skills are necessary for the development of ToM in early childhood. A longitudinal study of similarly aged autistic children supports this conclusion, in which the executive skills of 5‐year‐olds predicted ToM skills 3 years later, but not vice versa (Pellicano 2010).

To our knowledge, only one study has examined the relationship between EF and social cognition in autistic adults. Wilson et al. (2014) studied 89 male autistic adults (18–43 years old) using a comprehensive battery of social cognition and performance‐based EF measures, finding that all pairwise correlations between the two types of measures were weak. This suggests a null association between EF and social cognition in autistic adults, though correlations did not control for possible confounders such as age or IQ. Furthermore, the relationship between EF and emotion recognition (ER) is scarcely researched. Joseph and Tager‐Flusberg (Joseph and Tager–Flusberg 2004) speculate that EFs are more likely to be associated with ToM than they are with ER. This is based on reasoning that ER is a lower‐order and perceptual aspect of social cognition, while exercising ToM is conscious, effortful, and more likely to engage executive functions. There is some degree of support for this prediction. For instance, better performance‐based EF (planning and set‐shifting) predicted better ToM, but not better ER in a cross‐sectional sample of autistic children (Ozonoff et al. 1991), and better working memory in a sample of 9‐year‐old autistic children predicted ToM but not ER at one‐year follow‐up. Given that existing research focuses heavily on child cohorts, more research is needed to elucidate the relationship between EF and social cognition in autistic adults.

It is also important to consider how EF and social cognition explain variations in social responsiveness among autistic individuals. In children, higher levels of ER (Trevisan and Birmingham 2016), ToM (Bishop‐Fitzpatrick et al. 2017) and EF (Leung et al. 2016; Torske et al. 2018) are associated with lower levels of parent‐reported social responsiveness. There are fewer studies on autistic adults, but findings are similar. Autistic adults who recognize emotions better tend to exhibit less clinician‐reported and self‐reported social responsiveness (Humphreys et al. 2007; Kliemann et al. 2013) and those with greater baseline cognitive flexibility—a core domain of executive function—exhibit more adaptive behavior when assessed 3 years later (Berger et al. 2003). However, it is unclear whether EF and social cognition *jointly* predict social difficulties in autistic individuals or not. Growing evidence suggests that EF predicts social responsiveness independently of social cognition in autistic children (Pellicano 2013; Kenny et al. 2019), though some argue that EF predicts social responsiveness indirectly, via social cognition (Jones et al. 2018), and others have found that neither construct explains variation in social responsiveness more than the other (Joseph and Tager–Flusberg 2004; Cantio et al. 2016). Furthermore, we are aware of no existing study, which examines how EF and social cognition jointly contribute to social responsiveness in autistic adults.

### 1.1 Aims and Hypotheses

The aim of this study was to examine the relationship between social cognition and executive function and their influence on social functioning outcomes in autistic adults. The first aim of this study was to determine how executive function predicts social cognition. We hypothesized that better EF would predict better theory of mind, but not better emotion recognition in autistic adults. The second aim was to determine how EF and social cognition predict social difficulties in autistic adults. We predicted that better EF would be associated with lower levels of social responsiveness. We also predicted that better social cognition would be associated with lower levels of social responsiveness. Finally, we predicted that better social cognition would mediate the association between better EF and lower levels of social responsiveness.

## 2 Method

### 2.1 Participants

A total of 305 treatment‐seeking autistic participants presented to the Clinic for Autism and Neurodevelopment (CAN) Research at the Brain and Mind Centre via self‐referral, referral from a healthcare professional, or from collocated *Headspace* clinics. The sample consisted of 183 males and 122 females, ranging from 16 to 80 years old (*M* = 26.12, SD = 11.21). Participants were included if a formal diagnosis of ASD was indicated, as determined by onsite assessment (Autism Diagnostic Observation Schedule‐Second Edition (ADOS‐2) (Lord et al. 2012); or the Autism Diagnostic Interview Schedule‐Revised (Lord et al. 1994)). Participants were excluded if they demonstrated intellectual disability (IQ < 70 as determined by the Weschler Test of Adult Reading [WTAR] (Weschler 2001)), reported current substance dependence or suicidal intent. The University of Sydney Ethics Committee (research protocol No. 2013/352) approved this study. All participants provided informed written and verbal consent prior to assessment.

### 2.2 Measures

#### 2.2.1 General Intellectual Functioning

The Weschler Test of Adult Reading provides an estimate of full‐scale IQ, based on participants' ability to pronounce 50 words of increasing difficulty (Weschler 2001). The number of words read is converted to a standardized score, which is then converted to an estimate of full‐scale IQ.

#### 2.2.2 Social Cognition

The Reading the Mind in the Eyes Test (RMET) assesses lower‐order social cognition. It tests the ability to label emotions and metalize from 36 photographs of the human eye region (Baron‐Cohen et al. 2001). The Faux Pax Recognition Test (FPRT)assesses higher‐order social cognition. It tests whether participants can recognize and understand socially awkward interactions (Stone et al. 1998).

#### 2.2.3 Executive Functioning

Performance‐based EF was assessed using two standardized, neuropsychological tests. The Trail Making Test (TMT) assesses psychomotor speed and cognitive flexibility (Reitan and Wolfson 1985). Part B of the TMT (TMT‐B) is sensitive to task‐switching ability in neurotypical adults (Sánchez‐Cubillo et al. 2009) and is widely used to measure executive function in autistic adults (Geurts et al. 2009). As TMT‐B times were heavily left‐skewed, we applied a reciprocal transformation such that the transformed variable represented the rate at which the test was completed. The Controlled Oral Word Association Test (COWAT) assesses verbal fluency, requiring participants to generate as many words as possible belonging to a given category in one minute (Lezak et al. 2004). The Letters subtest (measuring phonemic fluency) and Animals subtest (measuring semantic fluency) were combined to yield a COWAT *Z* score.

Self‐reported executive functioning was measured using the Behavior Rating Inventory of Executive Function—Adult Version (BRIEF‐A) (Roth et al. 2005). The BRIEF‐A consists of 75 statements totaled into a Global Executive Composite (GEC) and consists of nine clinical subscales: Inhibit, Shift, Self‐Monitor, Emotional Control, Initiate, Plan/Organize, Working Memory, Organization of Materials, and Task Monitor. Henceforth, we refer to the BRIEF‐A as the BRIEF, unless otherwise stated. Raw scores are converted to age‐normed *T* scores, with higher scores indicating greater degrees of executive dysfunction. *T* scores ≥ 65 suggest clinically significant responsiveness on executive functioning.

#### 2.2.4 Social Responsiveness

Clinician‐rated social responsiveness was measured using the Autism Diagnostic Observation Schedule‐2 (ADOS‐2), a semi‐structured observational assessment used for diagnosing ASD in children and adults (Lord et al. 2012). The ADOS Calibrated Severity Score (CSS) was used to gauge the extent of social responsiveness, with higher scores indicating greater social responsiveness. The ADOS CSS algorithm incorporates scores on the Social Affect domain and the Restricted and Repetitive Behavior domains of the ADOS, and is a reliable indicator of symptom severity in autistic adults (Hus and Lord 2014).

Self‐reported social responsiveness was measured using the Social Responsiveness Scale 2 (SRS‐2) (Constantino and Gruber 2012). Items load onto five subscales: social “Awareness”, “Cognition”, “Communication”, “Motivation” and “Restricted Interests and Repetitive Behaviors.” The sum of the first four subscales forms a broader “Social Communication and Interaction Subscale (SCI)”, which formed the outcome variable for this study. Raw scores are converted into *T* scores, with the following ranges indicating the severity of social responsiveness: ≤ 59 *T*, normal; 60‐65 *T*, mild; 65–75 *T* moderate; ≥ 76 *T* severe. Henceforth, we refer to the SRS‐2 as the SRS, unless otherwise stated.

### 2.3 Analysis

Data cleaning, inspection, and descriptive analysis were conducted using SPSS Statistics, Version 27 (IBM 2020). Correlation, regression, and mediation analyses were conducted in R, Version 4.11 (R Foundation 2021a, 2021b). All social cognition, executive function, and social responsiveness measures were inspected for normality (Table S1).

Missing items on the BRIEF and SRS were handled according to their respective administrative manuals. Multiple imputation was used to handle scale‐level missingness on the BRIE, SRS, and all other variables. Twenty imputed datasets, with 10 iterations between each imputation, were generated using the R package ‘mice’. Values were imputed by predictive mean matching. Each imputed dataset was analyzed separately; results were then combined to produce pooled parameter estimates.

To examine the relationship between EF and social cognition, we used hierarchical regressions. EF scores were used to predict higher‐order and lower‐order social cognition scores in separate regressions. In Step 1, sex, age, and IQ were entered. In Step 2, TMT‐B and COWAT scores were entered. In Step 3, BRIEF scores were entered.

To determine the joint contributions of EF and social cognition to social responsiveness, we conducted several mediation analyses. We ran 12 separate mediations, corresponding to the 3 (EF: TMT‐B, COWAT, BRIEF GEC) × 2 (social cognition: RMET, FPRT) × 2 (social responsiveness: ADOS CSS, SRS‐2) permutations of models in which EF predicts social responsiveness, through social cognition.

**FIGURE 1:**
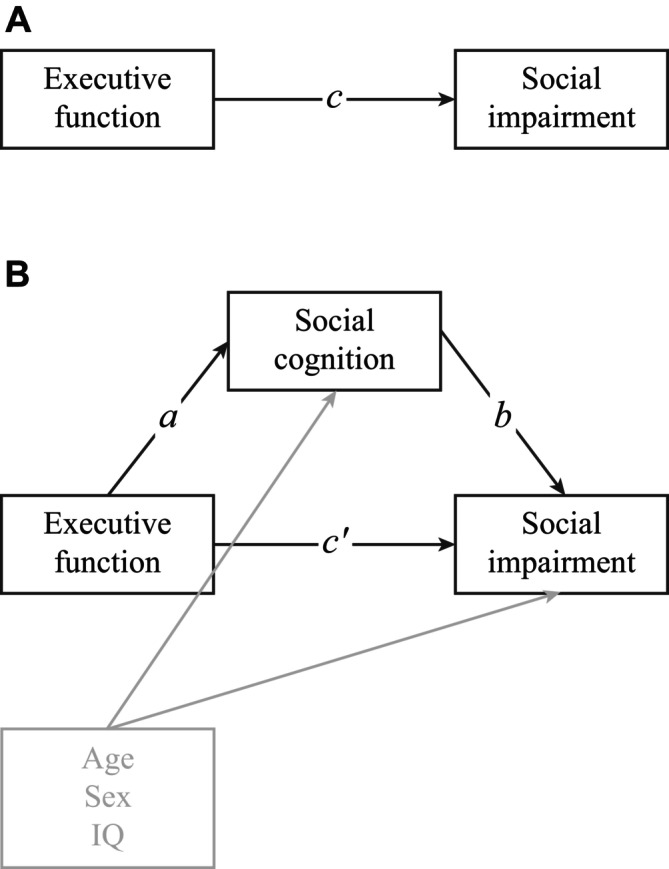
Details of the proposed mediation model between executive function, social cognition and social impairment. (A) *c* = *total effect*; how well executive function (EF) predicts social impairment, without considering mediators. (B) *a* = how well EF predicts social cognition. *b* = how well social cognition predicts social impairment, holding levels of EF constant. *c*′ = *direct effect*; how well EF predicts social impairment, holding levels of social cognition constant. a*b (the product of *a* and *b*) = *indirect effect*; how well EF predicts social impairment, with social cognition as the mediating variable. All paths are adjusted for the effects of age, sex, and IQ on social cognition and social impairment (shown in gray).

Figure 1 illustrates the generic mediation model and its associated paths. Each path arrow represents a separate regression, where variables at the base are predictors and variables at the tip are outcomes. The *effects* associated with each path are regression coefficients. In a model without a mediator, the effect of EF on social responsiveness is known as the *total effect* (see Figure 1A). A simple mediation model (Figure 1B) permits examination of the *direct effect* and *indirect effect* of an independent variable on a dependent variable, in the presence of a mediating variable. In our case, the *direct effect* refers to how well EF scores predict social responsiveness scores, when controlling for the levels of the mediator (social cognition). The *indirect effect* refers to how well EF scores predict social responsiveness scores, when the mediator (social cognition) is allowed to vary. The *indirect effect* is computed as the product of path *a* and path *b*, where *a* is how well executive function scores predict social cognition scores and *b* is how well social cognition predicts social responsiveness, while controlling for EF scores. According to Preacher and Hayes (Preacher and Hayes 2008), social cognition mediates the relationship between EF and social responsiveness if a*b differs significantly from zero.

## 3 Results

### 3.1 Missing Data

Overall, 316 out of 3355 (9.42%) data points were missing, with missingness ranging from 3.6% for IQ to 29.2% for BRIEF GEC scores.

### 3.2 Descriptive Statistics

Descriptive statistics on participant data and test scores are shown in Table 1. Summaries are based on multiply imputed datasets. See Table S2 for descriptive statistics on the original (non‐imputed) dataset. See Table S3 for correlations between measures of interest.

**TABLE 1:** Descriptive statistics for demographic, social cognition, EF and social outcome measures.

	*N*	Mean	SD	Range
Demographics				
Sex				
Male	183	—	—	—
Female	122	—	—	—
Age	305	26.12	11.21	16–80
IQ	305	110.01	12.45	71–129
Social cognition measures				
RMET	305	23.90	5.80	6–35
FPRT	305	32.49	7.01	7–40
Executive function measures				
COWAT Letters^a^	305	36.68	12.68	11–83
COWAT Animals^a^	305	21.22	6.24	8–42
COWAT *Z*	305	−0.02	0.90	−2.04‐3.18
TMT‐B (s)^a^	305	80.49	47.04	29–376
TMT‐B (s^−1^)	305	0.0153	0.0062	0.0027–0.0345
BRIEF GEC	305	69.84	12.25	38–99
BRIEF Inhibit	—	62.01	11.91	39–92
BRIEF Shift	—	70.36	12.64	38–87
BRIEF Emotional Control	—	62.10	11.89	38–86
BRIEF Self‐Monitor	—	61.92	13.75	37–90
BRIEF Initiate	—	70.46	12.30	37–92
BRIEF Working Memory	—	71.82	13.36	39–97
BRIEF Plan and Organize	—	68.26	12.80	39–94
BRIEF Task Monitor	—	66.57	12.44	36–95
BRIEF Org. of Materials	—	58.12	11.66	36–86
Social impairment measures				
ADOS CSS	305	5.57	1.74	3–10
SRS SCI	305	69.56	9.09	39–90

*Note*: COWAT *Z* refers to the score generated after standardizing and combining scores on COWAT Letters and COWAT Animals. TMT‐B (s^−1^) refers to the speed at which the TMT‐B is completed, computed by taking the reciprocal of time taken to complete TMT‐B (s). Statistics were computed from multiply imputed datasets.

Abbreviations: ADOS CSS, ADOS Calibrated Severity Score; COWAT, Controlled Oral Word Association Test; FPRT, Faux Pas Recognition Test; GEC, global executive composite; IQ, WTAR‐predicted IQ; RMET, reading the mind in the eyes test; SCI, Social Communication and Interaction subscale; TMT‐B, Trail Making Test Part B.

^a^ Measures not included in subsequent analyses.

### 3.3 Regression Analysis

Two hierarchical regressions were conducted to determine the ability of EF to predict ToM and ER. Regression coefficients of each variable and the increment in variance explained at each step are shown in Tables 2 and 3.

**TABLE 2:** Regressing RMET scores on executive functioning, controlling for demographics.

	*b*	SE_*b*_	β	$R^{2}$	$\Delta R^{2}$
Step 1	—	—	—	0.157	0.157***
Sex	0.105	0.653	0.009	—	—
Age	0.048	0.028	0.093	—	—
IQ	0.176***	0.029	0.377	—	—
Step 2	—	—	—	0.161	0.004
COWAT	0.396	0.447	0.062	—	—
TMT‐B	3.549	58.016	0.004	—	—
Step 3	—	—	—	0.166	0.005
BRIEF GEC	−0.030	0.031	−0.064	—	—

*Note*: *p < 0.05; **p < 0.01; ***p < 0.001.

Abbreviations: COWAT, Controlled Oral Word Association Test; GEC, Global Executive Composite; IQ, WTAR‐predicted IQ; RMET, Reading in the Mind in the Eyes Test; TMT‐B, Trail Making Test Part B.

**TABLE 3:** Regressing Faux Pas Recognition Test scores on executive functioning.

	*b*	SE_*b*_	β	$R^{2}$	$\Delta R^{2}$
Step 1	—	—	—	0.152	0.152***
Sex	1.239	0.780	0.087	—	—
Age	−0.006	0.035	−0.010	—	—
IQ	0.209***	0.034	0.371	—	—
Step 2	—	—	—	0.173	0.021*
COWAT	0.506	0.505	0.065	—	—
TMT‐B	141.433*	71.307	0.125	—	—
Step 3	—	—	—	0.174	0.001
BRIEF GEC	−0.015	0.035	−0.026	—	—

*Note*: *p < 0.05, **p < 0.01, ***p < 0.001.

Abbreviations: COWAT, Controlled Oral Word Association Test; FPRT, Faux Pas Recognition Test; GEC, Global Executive Composite; IQ, WTAR‐predicted IQ; TMT‐B, Trail Making Test Part B.

#### 3.3.1 Predicting Emotion Recognition

In the first step, age, sex and IQ were entered as demographic control variables. Together, they accounted for 15.7% of variance in RMET scores (*F*(3, 1249) = 15.879, *p* < 0.001). IQ was the only significant predictor of RMET score. When controlling for sex and age, each IQ point increase was associated with a 0.176 point increase in RMET score (SE_*b*_ = 0.029, *t*_73_ = 5.981, *p* < 0.001). In the second step, the performance‐based executive function measures (COWAT and TMT‐B) were entered. Neither measure significantly predicted RMET score, and together they did not explain additional variance in RMET score over and above the demographic variables (∆*R* ^2^ = 0.004, *F*(2, 903) = 0.496, *p* = 0.609). In the third step, BRIEF GEC score was entered. BRIEF GEC score did not account for additional variance in RMET score over and above the variables entered in the previous two steps (∆*R* ^2^ = 0.005, *F*(1, 78) = 0.925, *p* = 0.339). IQ remained a significant predictor of RMET score in each step.

#### 3.3.2 Predicting Theory of Mind

In the first step, age, sex and IQ were entered. Together, they accounted for 15.2% of variance in FPRT score (*F*(3, 1527) = 15.469, *p* = 0.001). IQ was the only significant predictor of FPRT score, such that each IQ point increase, when controlling for sex and age, corresponded to a 0.209 point increase in FPRT score (SE_*b*_ = 0.034, *t*_100_ = 6.091, *p* < 0.001). In the second step, the performance‐based executive function measures were entered. Together, they only explained an additional 2.1% of the variance in FPRT score, which was statistically significant (∆*R* ^2^ = 0.021, *F*(2, 1822) = 0.576, *p* = 0.034). Although the variance explained was very small, TMT‐B was a significant predictor of FPRT score (*b* = 141.433, SE_*b*_ = 71.307, *t*_215_ = 1.983, *p* = 0.049). In the third step, BRIEF GEC score was entered. BRIEF GEC score did not explain additional variance in FPRT score beyond the variance explained by the previous two steps (∆*R* ^2^ = 0.001, *F*(1, 146) = 0.177, *p* = 0.675). TMT did not remain as a significant predictor in the third step, while IQ remained a significant predictor in both the second and third step.

### 3.4 Mediation Analysis

**FIGURE 2:**
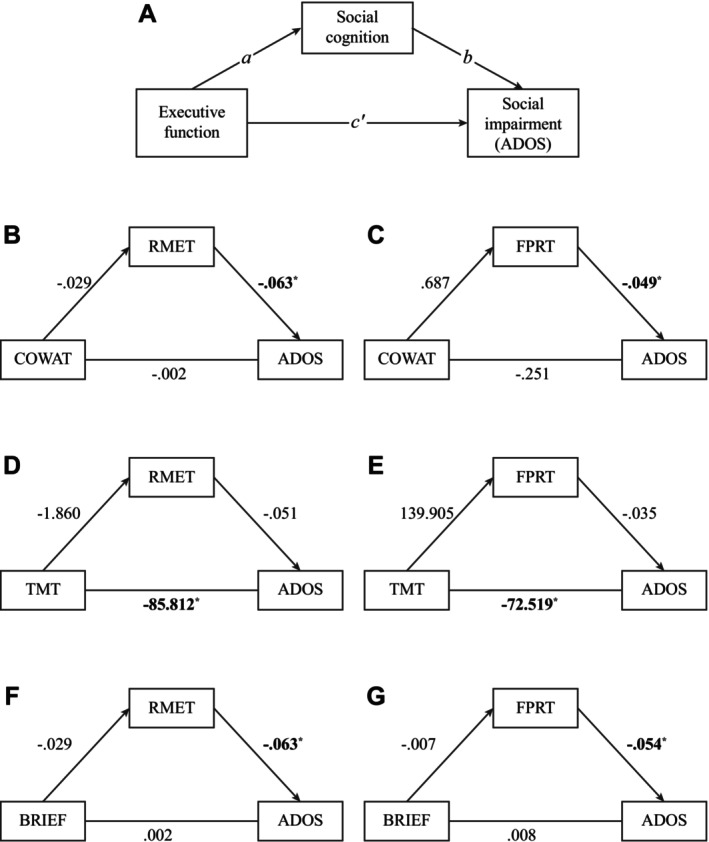
Mediation models with significant paths predicting ADOS scores. (A) Depiction of the generic mediation model, labeled with terms associated with each path. *Not shown*: All paths are adjusted for the effects of age, sex, and IQ (refer to Figure 1B). (B–E) Models predicting ADOS score which contain significant paths. Paths are unstandardized regression coefficients. * indicates a significant path. ADOS CSS, ADOS Calibrated Severity Score; COWAT, Controlled Oral Word Association Test; FPRT, Faux Pas Recognition Test; RMET, Reading the Mind in the Eyes Test; TMT, Trail Making Test Part B.

**FIGURE 3:**
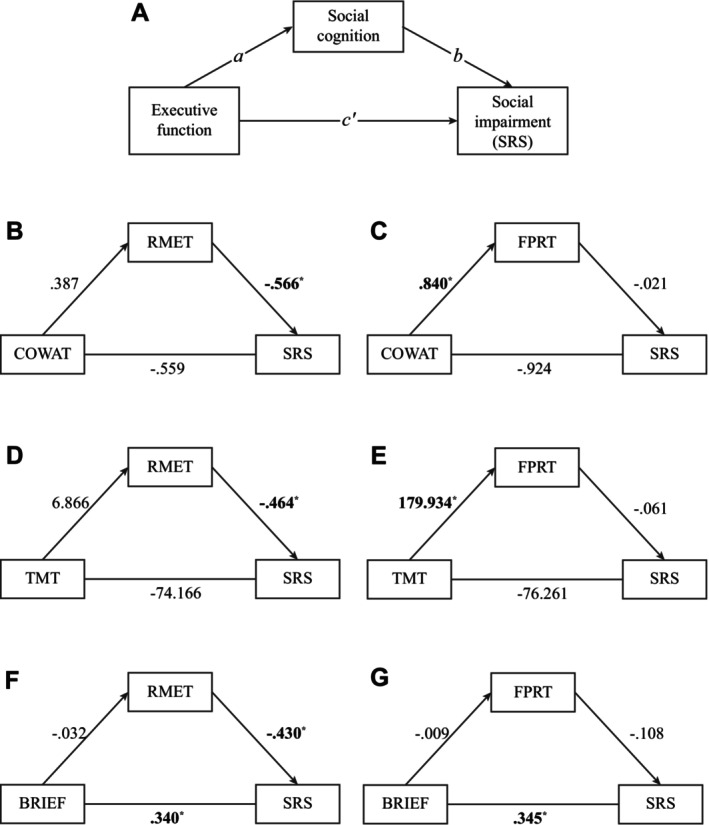
Mediation models with significant paths predicting SRS score. (A) Depiction of the generic mediation model, labeled with terms associated with each path. *Not shown*: all paths are adjusted for the effects of age, sex, and IQ (refer to Figure 1B). (B–D) Models predicting SRS score which contain significant paths. Paths are unstandardized regression coefficients. * indicates a significant path. COWAT, Controlled Oral Word Association Test; FPRT, Faux Pas Recognition Test; RMET, Reading the Mind in the Eyes Test; SRS, SRS Social Communication and Interaction subscale; TMT, Trail Making Test Part B.

Twelve simple mediation models were constructed to determine the paths between EF, social cognition, and social responsiveness. The social responsiveness variable was ADOS score in six of the models and SRS score in the remaining six. Models with significant path coefficients are shown in Figure 2 (ADOS models) and Figure 3 (SRS models). Details for all models can be found in Supplementary Table S6.

Of the models predicting ADOS scores, none contained a significant indirect effect (path a*b). Two models contained a significant direct effect (path *c’*). Completing the TMT faster predicted lower ADOS CSS when controlling for RMET score (*b* = −85.812, SE_*b*_ = 16.756, 95% CI = [−123.205, −60.111]) and when controlling for FPRT score (*b* = −72.519, SE_*b*_ = 16.393, 95% CI = [−105.099, −41.363]). Four of the models contained a significant path *b*. When controlling for COWAT performance, higher RMET score (*b* = −0.063, SE_*b*_ = 0.021, 95% CI = [−0.108, −0.021]) and higher FPRT scores (*b* = −0.049, SE_*b*_ = 0.015, 95% CI = [−0.076, −0.018]) was associated with lower ADOS CSS. A similar association emerged when controlling for BRIEF GEC score, such that having a higher RMET score (*b* = −0.063, SE_*b*_ = 0.021, 95% CI = [−0.108, −0.021]) and FPRT score (*b* = 0.054, SE_*b*_ = 0.015, 95% CI = [−0.082, −0.023]) predicted a lower ADOS CSS.

Of the models predicting SRS scores, none contained a significant indirect effect (path a*b). Two contained a significant direct effect (path *c'*). In particular, higher BRIEF GEC score predicted higher SRS score when controlling for RMET score (*b* = 0.340, SE_*b*_ = 0.056, 95% CI = [0.224, 0.441]) and when controlling for FPRT score (*b* = 0.345, SE_*b*_ = 0.054, 95% CI = [0.207, 0.437]). Three models contained a significant path *b*. Higher RMET score predicted lower SRS score when controlling for COWAT score (*b* = −0.466, SE_*b*_ = 0.119, 95% CI = [−0.704, −0.226]), when controlling for TMT completion speed (*b* = −0.464, SE_*b*_ = 0.120, 95% CI = [−0.671, −0.194]), and when controlling for BRIEF GEC score (*b* = −0.430, SE_*b*_ = 0.096, 95% CI = [−0.636, −0.257]). Two models contained a significant path *a*. A higher COWAT score predicted a higher FPRT score (*b* = 0.840, SE_*b*_ = 0.476, 95% CI = [0.000, 1.915]) and completing the TMT faster predicted a higher FPRT score (*b* = 179.934, SE_*b*_ = 77.784, 95% CI = [48.553, 359.559]).

Overall, none of the 12 models contained a significant *indirect effect*. Four models contained a significant *direct effect* (EF predicting social responsiveness), seven contained a significant path *b* (social cognition predicting social responsiveness). Two contained a significant path to *a* (EF predicting social cognition) but these latter two models did not predict social responsiveness.

## 4 Discussion

This study sought to examine the relationship between social cognition and EF and evaluate their influence on social functioning. We found that performance on both social cognition and EF tests predicted social responsiveness in autistic adults, but these effects are likely through separate pathways. A series of mediation models were conducted to determine the degree to which EF and social cognition explained social difficulties. For social cognition, emotion recognition and metalizing (Reading the Mind in the Eyes Test) and high‐order social cognition (Faux Pax Recognition Test) predicted social responsiveness. For EF, cognitive flexibility, but not verbal fluency, predicted social responsiveness in autistic adults. In sum, this exploratory study suggests that EF and social cognition are not strongly associated in autistic adults and supports the view that EF and social cognition are both clinically significant predictors of social difficulty, but through different pathways.

There has been extensive research demonstrating that autistic children and adults have lower scores on social cognition tests (Peñuelas‐Calvo et al. 2019). There have been no studies, however, exploring whether these scores have functional significance in terms of predicting outcomes on ADOS and SRS measures of social responsiveness. Findings here support the conclusion that social cognition scores do have prediction capacity for social outcomes. Performance on these tests has explanatory power for understanding social responsiveness, above and beyond IQ and measures of EF.

These findings build on work which indicates that social cognitive ability contributes to functional and social skills in autistic adults, albeit indirectly and via neurocognitive capacity (Sasson et al. 2020), and indicates that social cognitive ability may be an identifiable and measurable contributor to social outcomes in autistic adults. Further investigation of how social cognitive ability may predict social outcomes in autism would advance theoretical understanding of the distinct contribution of social cognition to social outcomes, independent of neurocognitive and executive functioning processes. Such work would also provide important insights into the potential utility of social cognition as a targeted focus for intervention.

Our study demonstrates that EF has an important and independent relationship with social difficulty in autistic adults. Among significant models, EF exhibited a direct effect on social responsiveness, without significant mediation by social cognition. This corroborates studies of autistic children, where performance‐based (Kenworthy et al. 2009) and parent‐reported EF (Leung et al. 2016; Torske et al. 2018) are well‐known predictors of social functioning. In addition, the lack of mediation by social cognition supports prior research in which better EF (regardless of ToM) longitudinally predicts less social responsiveness (Pellicano 2013; Kenny et al. 2019). We offer explanations for each instance where EF predicted social responsiveness. Firstly, our finding of a negative relationship between cognitive flexibility and social responsiveness could be attributed to the conversational nature of the ADOS Module 4. Engaging in conversation requires one to respond flexibly to one's partner in a specific social context. Difficulties exercising this skill likely translate to greater social difficulty (Geurts et al. 2009). Secondly, we observed positive associations between self‐reported EF and self‐reported social responsiveness in our sample. This could be because domains of EF measured by the BRIEF could map onto skills important for establishing and maintaining relationships with others. For instance, an autistic individual with a high BRIEF Initiate score may have challenges starting conversations, leading to poorer self‐reported social responsiveness. This idea was explored by Lieb and Bohnert (2017), who found that higher BRIEF Shift, Working Memory, and Inhibition scores were associated with poorer caregiver‐rated social responsiveness and poorer perceived friendship quality among autistic adolescents. Because we only examined how BRIEF global scores (not individual domain scores) predicted social responsiveness, further research on how individual domains of self‐reported EF predict social responsiveness in autistic adults is warranted.

In addition, our study is the largest to date to investigate the relationship between EF and social cognition in autistic adults. Contrary to our hypothesis, we found that EF either did not, or had a weak relationship, with both higher‐order (ToM) and lower‐order (ER) social cognition. This finding contrasts with the positive association between EF and socio‐cognitive ability observed in autistic children (Pellicano 2007; Pellicano 2010), but concurs with the single study of autistic adults in which no strong associations between cognitive variables were observed (Wilson et al. 2014). One possible explanation is that the relationship between EF and social cognition in autism changes across development. EF matures along a protracted trajectory for autistic individuals but remains reduced across the lifespan, relative to neurotypical individuals (Demetriou, Lampit, et al. 2018; Luna et al. 2007; O'Hearn et al. 2008). Hence, it could be that, as autistic children mature and become more aware of their executive challenges, they develop compensatory mechanisms (Livingston and Happé 2017). These compensatory mechanisms could rely on select aspects of EF or obviate the need for EF entirely. Alternatively, the child research relies more heavily on informant reports. Being an adult sample, relationships may be different when using self‐report measures. More research is needed to define the role of compensation in the cognitive development of autistic individuals and the differences between informant and self‐report measures.

Interestingly, we observed that the association between better EF and less social responsiveness was qualified by mode of assessment. Better performance‐based EF (cognitive flexibility) corresponded to less clinician‐observed social responsiveness (Figure 1B–E), while higher ratings of self‐reported EF corresponded to less self‐reported social responsiveness (Figure 2C,D). A potential explanation for this pattern could be high‐level similarities in the tasks being administered. According to Toplak et al. (Toplak et al. 2013), self‐report measures such as the BRIEF and SRS probe the beliefs of the participant, are typically completed without constraint, and engage the “reflective mind.” In contrast, tasks such as traditional EF tests and the ADOS are more concerned with the participant's performance, require them to perform under standardized constraints, and engage the “algorithmic mind.” These broad (and unintentional) similarities among tasks may explain our pattern of associations. The distinction between reflective and algorithmic tasks is also suggested by the fact that IQ was significantly (zero‐order) correlated with EF test and ADOS scores, *but not* BRIEF and SRS scores (Table S3). To address unintentional grouping of tasks, we encourage future research to include more measures and to combine them appropriately.

We note several limitations in our study. Firstly, we made no comparisons with a neurotypical control group, nor with other clinical groups. This protocol has, however, been used in a smaller autistic population, and all measures of EF and social cognition were shown to differentiate autistic and neurotypical adults (Pepper et al. 2018; Demetriou, Song, et al. 2018), although these measures are less likely to differentiate between different clinical groups related to neurodevelopment (Sadozai et al. 2024). Second, those with intellectual disability were excluded, meaning that our sample may not have fully captured the diversity of autistic features present in the autistic population. We also included a sample that reported relatively high levels of intellect. We note that we controlled for IQ in all of our mediation models. This sample is not, however, representative of all those assigned an autism diagnosis, and further research is required addressing relationships between these constructs in populations with lower IQ. Thirdly, there is debate in the field about the best measures of social cognition and EF, as well as those outcomes that are most relevant to social functioning and what is important to the autistic community. We chose these measures because they are widely regarded as suitable measures for the constructs identified and are also widely used in clinical trials and services. Previous work has shown their value in differentiating performance in autistic individuals from other populations, such as adults with social anxiety disorder and early psychosis (Pepper et al. 2018; Demetriou, Song, et al. 2018), though we encourage researchers to replicate our results with different measures. We also recognize that the ADOS has been criticized when it is used as a continuous severity measure, *even with the calibrated severity score*. The scales we chose to measure each construct were somewhat dissimilar to each other. To account for this, we ran multiple mediation models. Not all models revealed the same findings, and we drew conclusions by considering the paths, which reached statistical significance the most frequently. We note that the non‐significance of the model was also a frequent outcome. While our statistical methods and interpretations are appropriate, we acknowledge and emphasize the exploratory nature of this study. Our study adopted a cross‐sectional design, which limited our ability to draw directional conclusions. Future longitudinal studies are needed to evaluate this possibility. Finally, given that some of the tasks and relationships observed were between self‐report measures, we cannot rule out that reporter biases could underlie relationships between these measures.

In conclusion, our study highlights the importance of EF and social cognition as separate markers of social difficulty responsiveness in autistic adults. This study shows that, unlike studies in autistic children, EF and social cognition were unrelated in autistic adults. This observation highlights the need to consider lifelong trajectories of cognitive development and an urgent need to increase the evidence base in autistic adults. Our findings support EF and social cognition as a potentially useful marker of social responsiveness and a potential target for clinical assessment and intervention. Though a growing number of interventions are being developed to improve psychosocial functioning in autistic individuals (Pallathra et al. 2019), few are targeted toward adults, and even fewer are specifically designed to improve EF (Solomon et al. 2004). The lack of available evidence‐based supports for autistic adults is one likely factor behind the unique “services cliff” faced by these individuals.

## Ethics Statement

The University of Sydney Ethics Committee (research protocol No. 2013/352) approved this study.

## Conflicts of Interest

The authors declare no conflicts of interest.

## Supporting information


**Appendix S1.** Supplementary Tables.

## Data Availability

The data that support the findings of this study are available on request from the corresponding author. The data are not publicly available due to privacy or ethical restrictions.
