# Supplementary material for: Executive Function and Social Cognition Performance Predicts Social Difficulty for Autistic Adults
Source: Autism Res. 2025 Aug 1;18(9):1734–45. doi: 10.1002/aur.70090 (PMC12442527; doi:10.1002/aur.70090)
Supplement: Supplementary file 1 — Appendix S1. Supplementary Tables. [file AUR-18-1734-s001.docx]

**Table S1. Normality statistics for continuous variables**

|  | Shapiro-Wilk Normality Test | | |  | Skewness | |  | Kurtosis | |
| --- | --- | --- | --- | --- | --- | --- | --- | --- | --- |
| Variable | *W* | df | Sig. |  | Skewness | Skewness *Z* |  | Kurtosis | Kurtosis *Z* |
| Age | .729 | 305 | <.001 |  | 2.402 | 17.212* |  | 6.556 | 23.561* |
| IQ | .927 | 294 | <.001 |  | -1.123 | -7.903* |  | 1.562 | 5.512* |
| RMET | .948 | 272 | <.001 |  | -0.855 | -5.791* |  | 0.622 | 2.114* |
| FPRT | .882 | 277 | <.001 |  | -1.195 | -8.165* |  | 1.135 | 3.890* |
| COWAT Letters | .977 | 278 | <.001 |  | 0.585 | 4.003* |  | 0.499 | 1.713 |
| COWAT Animals | .988 | 278 | .022 |  | 0.336 | 2.302* |  | 0.267 | 0.918 |
| TMT-B | .981 | 282 | .001 |  | 0.471 | 3.248* |  | -0.024 | -0.084 |
| BRIEF GEC | .991 | 215 | .210 |  | -0.129 | -0.776 |  | -0.427 | -1.293 |
| ADOS CSS | .939 | 305 | <.001 |  | 0.261 | 1.867 |  | -0.652 | -2.342* |
| SRS SCI | .993 | 227 | .320 |  | -0.140 | -0.865 |  | 0.008 | 0.025 |

**Normality tests, skewness and kurtosis of continuous variables.** *Z* scores were calculated by dividing skewness and kurtosis statistics by their associated standard error. * indicates a statistically significant skewness or kurtosis (|*Z*| > 1.96). IQ = WTAR-predicted IQ. RMET = Reading the Mind in the Eyes Test. FPRT = Faux Pas Recognition Test. TMT-B = Trail-making test Part B (seconds). COWAT = Controlled Oral Word Association Test. GEC = Global Executive Composite. ADOS CSS = ADOS Calibrated Severity Score. SCI = Social Communication and Interaction subscale.

Table S2. Descriptive statistics of the original sample

|  | | N | Mean | *SD* | Range |
| --- | --- | --- | --- | --- | --- |
| Demographics | |  |  |  |  |
| Sex | Male | 183 |  |  |  |
|  | Female | 122 |  |  |  |
| Age | | 305 | 26.12 | 11.21 | 16-80 |
| IQ | | 294 | 110.10 | 12.37 | 71-129 |
| Social cognition measures | | | | | |
| RMET | | 272 | 24.01 | 5.72 | 6-35 |
| FPRT | | 277 | 32.71 | 6.89 | 7-40 |
| Executive function measures | | | | | |
| COWAT Letters | | 278 | 37.01 | 12.60 | 11-83 |
| COWAT Animals | | 278 | 21.37 | 6.18 | 8-42 |
| COWAT *Z* | | 278 | 0.00 | 0.90 | -2.04-3.17 |
| TMT-B (seconds) | | 282 | 78.04 | 43.37 | 29-376 |
| TMT-B (seconds^-1^) | | 282 | 0.0155 | 0.0061 | .0027-.0345 |
| BRIEF GEC | | 215 | 69.46 | 12.79 | 38-99 |
| BRIEF Inhibit | |  | 61.95 | 12.14 | 36-92 |
| BRIEF Shift | |  | 70.14 | 12.91 | 39-91 |
| BRIEF Emotional Control | |  | 61.95 | 12.06 | 38-86 |
| BRIEF Self-Monitor | |  | 61.90 | 13.80 | 37-90 |
| BRIEF Initiate | |  | 70.19 | 12.45 | 37-91 |
| BRIEF Working Memory | |  | 71.63 | 13.58 | 39-97 |
| BRIEF Plan and Organize | |  | 67.96 | 13.04 | 39-94 |
| BRIEF Task Monitor | |  | 66.37 | 12.81 | 36-95 |
| BRIEF Org. of Materials | |  | 58.00 | 11.92 | 36-86 |
| Social impairment measures | |  |  |  |  |
| ADOS CSS | | 305 | 5.57 | 1.74 | 3-10 |
| SRS SCI | | 227 | 69.66 | 9.24 | 39-90 |

**Descriptive statistics for social cognition and executive function measures.** IQ = WTAR-predicted IQ. RMET = Reading the Mind in the Eyes Test. FPRT = Faux Pas Recognition Test. COWAT = Controlled Oral Word Association Test. TMT-B = Trail Making Test Part B. GEC = Global Executive Composite. ADOS CSS = ADOS Calibrated Severity Score. SCI = Social Communication and Interaction subscale. COWAT *Z* refers to the score generated after standardising and combining scores on COWAT Letters and COWAT Animals. TMT-B (seconds^-1^) refers to the speed at which the TMT-B is completed, computed by taking the reciprocal of time taken to complete TMT-B (seconds).

Table S3. Correlations between continuous measures

|  | Sex | Age | IQ | COWAT | TMT-B | BRIEF GEC | RMET | FPRT | ADOS |
| --- | --- | --- | --- | --- | --- | --- | --- | --- | --- |
| Age | 0.007 |  |  |  |  |  |  |  |  |
| IQ | 0.113 | 0.089 |  |  |  |  |  |  |  |
| COWAT | 0.118^*^ | 0.128^*^ | 0.418^***^ |  |  |  |  |  |  |
| TMT-B | 0.144^*^ | 0.119^*^ | 0.352^***^ | 0.437^***^ |  |  |  |  |  |
| BRIEF GEC | 0.114 | -0.015 | -0.039 | -0.044 | -0.075 |  |  |  |  |
| RMET | 0.045 | 0.135^*^ | 0.343^***^ | 0.205^**^ | 0.129^*^ | -0.085 |  |  |  |
| FPRT | 0.125^*^ | 0.074 | 0.341^***^ | 0.251^***^ | 0.266^***^ | -0.037 | 0.333^***^ |  |  |
| ADOS CSS | -0.192^***^ | -0.180^**^ | -0.159^**^ | -0.219^***^ | -0.321^***^ | 0.023 | -0.218^***^ | -0.214^***^ |  |
| SRS | 0.074 | 0.018 | -0.057 | -0.130 | -0.039 | 0.485^***^ | -0.261^***^ | -0.081 | 0.155^*^ |

Spearman correlations between key measures. IQ = WTAR-predicted IQ. COWAT = Controlled Oral Word Association Test. TMT-B = Trail Making Test Part B. GEC = Global Executive Composite. RMET = Reading the Mind in the Eyes Test. FPRT = Faux Pas Recognition Test. ADOS CSS = ADOS Calibrated Severity Score. SRS = SRS Social Communication and Interaction subscale. * *p* < .05, ** *p* < .01, *** *p* < .001.

Table S4. Exploratory analysis: BRIEF subscales predicting RMET scores.

| Subscale | *b* | *SE_b_* | $\beta$ | $\Delta R^{2}$ | $\Delta F$ | sig. $\Delta F$ |
| --- | --- | --- | --- | --- | --- | --- |
| Inhibit | -.045 | .034 | -.093 | .010 | *F*(1,189) = 1.770 | .191 |
| Shift | -.017 | .032 | -.038 | .003 | *F*(1,64) = .298 | .588 |
| Emotional Control | -.022 | .040 | -.045 | .005 | *F*(1,30) = .286 | .597 |
| Self-Monitor | -.104^**^ | .032 | -.245 | .059 | *F*(1,33) = 10.389 | .003 |
| Initiate | -.007 | .029 | -.015 | .001 | *F*(1,142) = .059 | .808 |
| Working Memory | .002 | .027 | .004 | .001 | *F*(1,149) = .005 | .946 |
| Plan and Organize | -.029 | .029 | -.063 | .005 | *F*(1,112) = 1.007 | .318 |
| Task-Monitor | .000 | .032 | .000 | .001 | *F*(1,63) = .000 | .993 |
| Organization of Materials | .010 | .033 | .020 | .002 | *F*(1,71) = .084 | .772 |

Individual BRIEF subscales were entered in Step 3, after sex, age and IQ (in Step 1) and COWAT, TMT-B (in Step 2). $\Delta R^{2}$ = additional variance explained by the subscale, over and above the variance explained by the predictors included in Step 2. $\Delta F$ = *F*-statistic for $\Delta R^{2}$. sig. $\Delta F$ = statistical significance of $\Delta F$. * *p* < .05, ** *p* < .01, *** *p* < .001.

Table S5. Exploratory analysis: BRIEF subscales predicting FPRT scores

| Subscale | *b* | *SE_b_* | $\beta$ | $\Delta R^{2}$ | $\Delta F$ | sig. $\Delta F$ |
| --- | --- | --- | --- | --- | --- | --- |
| Inhibit | -.018 | .039 | -.031 | .002 | *F*(1,78) = .217 | .642 |
| Shift | .001 | .034 | .002 | .001 | *F*(1,226) = .002 | .969 |
| Emotional Control | -.086^*^ | .039 | -.147 | .020 | *F*(1,106) = 5.018 | .027 |
| Self-Monitor | -.073 | .034 | -.143 | .021 | *F*(1,66) = 4.491 | .038 |
| Initiate | .031 | .036 | .054 | .004 | *F*(1,100) = .715 | .400 |
| Working Memory | .033 | .035 | .063 | .005 | *F*(1,70) = .901 | .346 |
| Plan and Organize | -.004 | .034 | -.007 | .001 | *F*(1,147) = .011 | .915 |
| Task-Monitor | -.006 | .033 | -.010 | .001 | *F*(1,218) = .029 | .866 |
| Organization of Materials | .018 | .042 | .030 | .003 | *F*(1,52) = .188 | .667 |

Individual BRIEF subscales were entered in Step 3, after sex, age and IQ (in Step 1) and COWAT, TMT-B (in Step 2). $\Delta R^{2}$ = additional variance explained by the subscale, over and above the variance explained by the predictors included in Step 2. $\Delta F$ = *F*-statistic for $\Delta R^{2}$. sig. $\Delta F$ = statistical significance of $\Delta F$. * *p* < .05, ** *p* < .01, *** *p* < .001.

**Table S6. Mediation analysis: all path coefficients, standard errors and confidence intervals**

|  | Model | *a* | *b* | *c* | *c'* | *a*b* |
| --- | --- | --- | --- | --- | --- | --- |
| 1. | COWAT 🡪 RMET 🡪 ADOS CSS | -.029 (.029) [-.083, .031] | -.063 (.021) **[-.108, -.021]** | .004 (.008) [-.015, .019] | .002 (.008) [-.016, .017] | .002 (.002)  [-.002, .007] |
| 2. | COWAT 🡪 FPRT 🡪 ADOS CSS | .687 (.501) [-.573, 1.483] | -.049 (.015) **[-.076, .-.018]** | -.284 (.133) **[-.519, -.013]** | -.251 (.129) [-.488, .003] | -.034 (.028) [-.095, .014] |
| 3. | TMT 🡪 RMET 🡪 ADOS CSS | -1.860 (55.765) [-117.823, 74.613] | -.051 (.020) [-.081, .001] | -85.879 (17.171) **[-122.203, -57.355]** | -85.812 (16.756) **[-123.205, -60.111]** | .023 (3.592) [-4.655, 8.416] |
| 4. | TMT 🡪 FPRT 🡪 ADOS CSS | 139.905 (73.012) [-14.573, 256.769] | -.035 (.015) [-.058, .006] | -77.386 (16.870) **[-105.911, -40.194]** | -72.519 (16.393) **[-105.099, -41.363]** | -4.868 (4.184) [-12.276, 1.617] |
| 5. | BRIEF 🡪 RMET 🡪 ADOS CSS | -.029 (.029) [-.084, .031] | -.063 (.021) **[-.108, -.021]** | .004 (.008) [-.015, .019] | .002 (.008) [-.016, .0517] | .002 (.002) [-.002, .007] |
| 6. | BRIEF 🡪 FPRT 🡪 ADOS CSS | -.007 (.037) [-.069, .078] | -.054 (.015) **[-.082, -.023]** | .008 (.008) [-.005, .025] | .008 (.008) [-.004, .025] | .000 (.002) [-.005, .004] |

**Path coefficients of mediation models predicting ADOS CSS.** COWAT = Controlled Oral Word Association Test. RMET = Reading the Mind in the Eyes Test. ADOS CSS = ADOS Calibrated Severity Score. FPRT = Faux Pas Recognition Test. TMT = Trail Making Test Part B. *c* = *total effect* of executive function on ADOS score. *c' = direct effect* of executive function on ADOS score. *a*b* = *indirect effect* of executive function on ADOS score, through social cognition. See Figure 1 or Methods for definitions of other effects. Path coefficients and their confidence intervals were estimated through 1000 bootstrap samples. 95% bias-corrected confidence intervals (BC CIs) were generated for each path coefficient. Paths are unstandardised regression coefficients, adjusted for the effects of sex, age and IQ. Paths are reported as *Estimate* (*standard error*) [*lower 95% BC CI, upper 95% BC CI*]. 95% BC CIs not containing zero are bolded.

|  | Model | *a* | *b* | *c* | *c'* | *a*b* |
| --- | --- | --- | --- | --- | --- | --- |
| 7. | COWAT 🡪 RMET 🡪 SRS | .384 (.377) [-.292, 1.166] | -.466 (.119) **[-.704, -.226]** | -.741 (.761) [-1.904, 1.074] | -.559 (.736) [-1.706, 1.170] | -.182 (.197) [-.676, .091] |
| 8. | COWAT 🡪 FPRT 🡪 SRS | .840 (.476) **[.000, 1.915]** | -.021 (.103) [-.181, .212] | -.945 (.734) [-2.155, .726] | -.924 (.735) [-2.150, .807] | -.021 (.091) [-.212, .726] |
| 9. | TMT 🡪 RMET 🡪 SRS | 6.866 (56.727) [-123.768, 100.560] | -.464 (.120) **[-.671, -.194]** | -74.166 (112.143) [-278.116, 150.703] | -71.479 (110.536) [-272.396, 141.297] | -2.687 (28.404) [-51.637, 58.420] |
| 10. | TMT 🡪 FPRT 🡪 SRS | 179.934 (77.784) **[48.553, 359.559]** | -.061 (.110) [-.265, .166] | -86.269 (113.632) [-322.837, 124.869] | -76.261 (118.397) [-332.133, 136.557] | -10.008 (20.603) [-56.626, 24.307] |
| 11. | BRIEF 🡪 RMET 🡪 SRS | -.032 (.028) [-.102, .016] | -.430 (.096) **[-.636, -.257]** | .354 (.056) **[.237, .447]** | .340 (.056) **[.224, .441]** | .014 (.013) [-.005, .055] |
| 12. | BRIEF 🡪 FPRT 🡪 SRS | -.009 (.037) [-.072, .080] | -.108 (.091) [-.392, .018] | .346 (.054) **[.207, .437]** | .345 (.054) **[.207, .437]** | .001 (.004) [-.004, .015] |

**Path coefficients of mediation models predicting SRS scores.** SRS = SRS Social Communication and Interaction subscale. Refer to the caption of the table above.
